# Rutin ameliorates oxidative stress and preserves hepatic and renal functions following exposure to cadmium and ethanol

**DOI:** 10.1080/13880209.2017.1387575

**Published:** 2017-10-12

**Authors:** Sunny O. Abarikwu, Rex-Clovis Njoku, Chiamaka J. Lawrence, Iniobong A. Charles, Jude C. Ikewuchi

**Affiliations:** Department of Biochemistry, University of Port Harcourt, Choba, Nigeria

**Keywords:** Serum, antioxidant, liver, kidney, phytochemicals

## Abstract

**Context:** Rutin (RUT) is an antioxidant flavonoid with well-known metal chelating potentials.

**Objective:** This study was designed to evaluate the protective effects of RUT against cadmium (Cd) + ethanol (EtOH)-induced hepatic and renal toxicity in rats.

**Materials and methods:** Wistar rats were treated with Cd (50 mg/kg) alone or in combination with EtOH (5 mg/kg) and RUT (25, 50 and 100 mg/kg) for 15 days. After treatment, the liver, kidney and serum were removed for biochemical assays by spectrophotometric methods.

**Results:** Serum, hepatic and renal malondialdehyde (MDA) levels were highest in the Cd + EtOH group and lowest in Cd + EtOH animals co-treated with the highest dose of RUT (2.98 ± 0.34, 10.08 ± 2.32, 4.99 ± 1.21 vs. 1.69 ± 0.33, 6.13 ± 0.28, 3.66 ± 1.12 μmol MDA/mg protein, respectively). The serum level of Cd was increased in the Cd + EtOH treated animals compared to Cd + EtOH animals co-treated with 100 mg/kg RUT (2.54 ± 0.08 vs. 1.28 ± 0.04 ppm). Furthermore, RUT at the highest dose protected against Cd + EtOH-induced elevation of bilirubin and uric acid levels as well as activities of lactate dehydrogenase and γ-glutamyl transferase (62.86 ± 2.74 vs. 122.52 ± 6.35 µmol/L; 1.77 ± 0.35 vs. 3.23 ± 0.55 mmol/L; 9.56 ± 1.22 vs. 16.21 ± 1.64 U/L; 288.92 ± 40.12 vs. 159.8 ± 18.01 U/L). The histo-pathological changes in the liver and kidney were also reduced in the Cd + EtOH animals co-treated with RUT in a dose-dependent manner.

**Discussion and conclusion:** RUT protected against the combined effects of Cd + EtOH on hepatic and renal functions and improved the antioxidant defence system in the blood.

## Introduction

Concurrent chemical exposures are frequent and individual chemical metabolism and excretion may be significantly influenced by interaction of mixtures (Lee et al. 2011). Modern toxicology seeks to unravel the adverse effects associated with simultaneous exposure to a variety of chemical mixtures. Synergistic interactions between xenobiotics and their potential health risk are underpinning recent toxicological studies (Jurczuk et al. 2004).

Cadmium (Cd) is a ubiquitous environmental pollutant that is used extensively in electroplating, plastics, paints, alloys and storage batteries (Brus et al. 1995). Long-term exposure to Cd has been implicated in organ damage and dysfunction in several experimental models (Sharara et al. 1998). The liver, kidney, lung and testis are susceptible to Cd-induced damage (Manca et al. 1991; Abarikwu et al. 2013). There is sufficient evidence in humans to classify Cd and Cd compounds as carcinogenic substances (Sadik 2008).

It well known that toxicity of xenobiotics can be influenced by biological and chemical factors, e.g., alcohol intake. More so, people exposed to work place pollutants consume alcoholic beverages chronically or occasionally (Brzóska et al. 2013). Simultaneous exposure to EtOH and Cd provoked injuries in liver, kidney, testis and brain of experimental animals (Flora and Tandon 1987; Sharma et al. 1991; Brzóska et al. 2013). Ethanol also enhances the carcinogenicity, mutagenicity, hepatotoxicity and testicular toxicity of various pollutants (Flora and Tandon 1987; Sharma et al. 1991). Ethanol may enhance Cd-induced oxidative stress by elevating the formation of free radicals and reactive oxygen species and by altering the activities of endogenous antioxidants (Sharma et al. 1991). The fact that EtOH increased the vulnerability of mammalian experimental models e.g. rats to the toxicity of Cd is also supported from findings on morphological studies (Brzóska et al. 2003). Because of their locations, structures and involvement in processing and removal of foreign compounds and waste products, the kidney and liver are vulnerable to toxic effects of Cd accumulation, EtOH biotransformation and environmental chemicals (Lock and Reed 1998; Jurczuk et al. 2004; Irving and Elfarra 2012).

Contemporary nutritional and therapeutic research focuses on the beneficial effects of dietary phytochemicals, such as flavonoids. Food bioactive compounds with antioxidant properties have been used against Cd-induced toxicity (Nazima et al. 2014). Of these phytochemicals, rutin (RUT), a citrus flavonoid, possess antioxidant, anti-allergic, anti-inflammatory, anti-angiogenic and antiviral properties (Nafees et al. 2015). Our previous studies and those of others have demonstrated the protective effects of RUT in different experimental models (Abarikwu et al. 2012; Aruna et al. 2014; Nafees et al. 2015). The present study, therefore, attempts to evaluate the antioxidant protective effects of RUT on the liver and kidney and its metal chelating potentials in rats treated with EtOH and Cd.

## Materials and methods

### Chemicals

Rutin, cadmium, and absolute ethanol (99.8%) were purchased from Sigma-Aldrich Chemical Company (St. Louis, MO). All other chemicals were of analytical grade.

### Treatment design and collection of samples

Thirty inbred male Wistar rats weighing 148.67 ± 10.43 g were divided into six experimental groups of five animals each. The animals were housed in metal cages placed in a well-ventilated condition, maintained on a 12 h photoperiod, provided with standard rat diet and water *ad libitum*. One week was allowed prior to the start of experiment for the animals to acclimatize to the surroundings. Group I received normal saline (control). Group II received CdCl_2_ (50 mg/kg body wt. dissolved in normal saline and administered by oral gavage throughout the study). Group III animals were administered Cd + EtOH (50 mg/kg body weight of CdCl_2_ and 5 g/kg body weight, 50% v/v of EtOH) by oral gavage. The solution containing Cd was administered first followed immediately by the administration of the mixture containing EtOH. Groups IV, V, and VI were pretreated with RUT (dissolved in normal saline) by oral gavage at doses 25, 50, or 100 mg/kg body weight, respectively, for one week prior to start of experiment, and thereafter both RUT and Cd + EtOH were administered throughout the two-week duration of study. The treatment of the chemicals to all animals was done three times weekly. The dose of RUT and EtOH used in this study was chosen based on previous studies that reported the antioxidant property of RUT (Abarikwu et al. 2013) and the capacity for Cd and EtOH to induce oxidative stress in the liver and kidney of rats (Jurczuk et al. 2004). The ethics regulation outlined in the Guide for the Care and Use of Laboratory Animals prepared by the National Academy of Science were followed throughout the study. At the end of the experiment, the animals were sacrificed by cervical dislocation. Blood samples were collected via cardiac puncture and the separated serum was used for assay of biochemical markers of oxidative stress as well as markers of liver and renal damage. Hepatic and renal tissues of experimental rats were homogenized in ice-cold 0.1 M Tris-HCl buffer (pH 7.4) to produce 10% homogenate. The homogenate was centrifuged at 10,000 *g*, at 4 °C for 15 min and the supernatant was separated to measure biochemical parameters of oxidative stress.

### Measurement of lipid peroxidation

The quantification of malondialdehyde (MDA) as marker of lipid peroxidation was estimated spectrophotometrically using thiobarbituric acid for colour development (Ohkawa et al. 1979) at 532 nm. TBARS in the samples were extrapolated using 1, 1, 3, 3-tetraethoxypropane as standard. The result was expressed as micromoles per milligram of protein. Protein concentration was determined by the method of Lowry et al. (1951).

### Estimation of reduced glutathione (GSH) level and activities of antioxidant defence enzymes

The GSH level was determined with a spectrophotometer at 412 nm using 5, 5′-dithiobis-(2-nitrobenzoic acid) for colour development (Sedlak and Lindsay 1968). The results were expressed as micromoles per mg protein. The activity of glutathione peroxidase (GSH-Px) was estimated with a spectrophotometer at 412 nm as previously described by Rotruck et al. (1973). The enzyme activity was expressed as units/mg protein. The activity of glutathione *S-*transferase (GST) was measured in a spectrophotometer at 340 nm using 1-chloro-2, 4-dinitrobenzene as a substrate (Habig et al. 1974). Superoxide dismutase (SOD) was determined spectrophotometrically at 480 nm using freshly prepared epinephrine (0.01%) as substrate (Misra and Fridovich 1972). The results were expressed as units/mg protein. Catalase (CAT) activity was measured as described by Clairborne (1995). The rate of degradation of H_2_O_2_, the substrate of the enzyme was calculated using 43.59 M^−1 ^cm^−1^ as the molar extinction coefficient of H_2_O_2_ at 240 nm. One unit of CAT activity equals the amount of protein that converts 1 mmol H_2_O_2_ per minute to H_2_O to O_2_. The enzyme activity was expressed as µmole H_2_O_2_/mg protein.

### Determination of biochemical markers of hepatic and renal functions

The activities of alkaline phosphatase (ALP), acid phosphatase (ACP), aspartate aminotransferase (AST), alanine aminotransferase (ALT), gamma-glutamyl transferase (GGT), and lactate dehydrogenase (LDH) as well as uric acid, cholesterol (CHOL) and bilirubin levels were assayed in the serum using Randox commercial kits (RANDOX Laboratories Ltd., Crumlin, UK) with an autoanalyzer (LabTech, RE 1201007), following the instructions provided by the manufacturers.

### Measurement of Cd concentration

Serum samples were digested with concentrated nitric acid (Merck, Darmstadt, Germany, 65%) at 120 °C. When fumes were white and the solution was completely clear, the samples were cooled to room temperature and the tubes were filled to 5 mL with ultra-pure water. All samples were analyzed to determine Cd using flame atomic absorption spectrometry. Samples were analyzed in triplicate and the variation in coefficient was usually less than 10%. Concentration of Cd was expressed as micrograms per litre in plasma.

### Histopathological examination

At necropsy, one kidney from three rats per experimental group and a portion of the liver of three rats per each experimental group were fixed with 10% buffered formalin. After 48 h, formalin-fixed tissues were embedded in paraffin wax according to the routine procedure, and 5 µm thick sections were cut with a rotary microtome. The sections were stained with hematoxylin and eosin for light microscopic examination.

### Statistical analysis

Data were expressed as mean ± SD, and the statistical analysis was performed using one-way analysis of variance followed by Dunnett’s *post hoc* test. The analysis was carried out with SPSS Statistics 17.0 (SPSS Inc., Chicago, IL). Significance was set at *p* < 0.05.

## Results

### Body and organ weights of Cd–EtOH intoxicated rats pretreated with RUT

All animals showed increased body weights compared to their initial weights. The differences in the weights were not statistically significant across all groups. The absolute and relative weights of the kidney of Cd, Cd + EtOH and 25 RUT + Cd + EtOH treated rats were decreased (*p* < 0.05) compared to control values. The decreased relative weight of the kidney was restored to the control values when the dose of RUT was increased to 50 and 100 mg/kg body wt. The animals in Cd + EtOH, 25 RUT + Cd + EtOH groups had decreased absolute and relative weights of the liver compared to Cd and control groups (*p* < 0.05). Administration of RUT (50, 100 mg/kg body wt.) to Cd + EtOH treated rats increased the absolute and relative weights of liver of animals toward control values (Table 1).

**Table 1.** Effect of rutin (RUT) on body weight, absolute and relative organ weights of the liver and kidney of rats after co-exposure to cadmium and ethanol (Cd + EtOH).

	Absolute (g)		Absolute (g)		Body weights (g)	
Groups	Liver	Relative (g/100 gm)	Kidney	Relative (g/100 gm)	Initial	Final
Control	6.91 ± 1.14^a^	3.84 ± 0.63^a^	0.96 ± 0.02^a^	0.53 ± 0.01^a^	148.34 ± 21.82	187.8 ± 19.58
Cd	6.35 ± 0.38^a^	3.34 ± 0.17^a^	0.79 ± 0.04^b^	0.36 ± 0.02^b^	165.3 ± 9.75	191.8 ± 11.73
Cd + EtOH	3.41 ± 0.97^b^	1.77 ± 0.49^b^	0.76 ± 0.09^b^	0.39 ± 0.05^b^	147.8 ± 15.85	178.3 ± 16.37
25 RUT + Cd + EtOH	3.04 ± 0.14^b^	1.84 ± 0.1^b^	0.72 ± 0.01^b^	0.42 ± 0.02^b^	139.58 ± 18.02	175.8 ± 15.14
50 RUT + Cd + EtOH	5.71 ± 0.42^a^	3.49 ± 0.29^a^	0.74 ± 0.04^b^	0.51 ± 0.03^a^	154.54 ± 14.20	185.8 ± 14.44
100 RUT + Cd + EtOH	6.59 ± 0.73^a^	3.68 ± 0.17^a^	1.06 ± 0.07^a^	0.59 ± 0.04^a^	136.42 ± 21.44	178.88 ± 15.03

Data are presented as the mean ± SD (*n* = 5). Values with different superscripts are significantly different from each other^.^ (*p* < 0.05).

### Serum Cd concentration and activities of hepatic and renal marker enzymes

Serum Cd levels were significantly increased in Cd and Cd + EtOH treated animals as compared to control (Table 2). Administration of RUT (25, 50, 100 mg/kg body wt.) to the Cd + EtOH treated animals decreased Cd level by 26.77, 31.89, and 49.61%, respectively, when compared to the Cd + EtOH-treated animals. Treatment with Cd or in combination with EtOH significantly increased (*p* < 0.05) the activities of GGT, LDH, ALP and ACP compared to control values. The activities of AST and ALT were statistically unchanged (*p* > 0.05) across all groups. The activities of GGT, LDH and ACP were significantly decreased (*p* < 0.05) in 50 RUT + Cd + EtOH and 100 RUT + Cd + EtOH-treated animals compared to Cd and Cd + EtOH groups. The high-dose RUT (100 mg/kg body wt.) but not the intermediate dose (50 mg/kg body wt.) restored the activity of ALP to near control values (Table 2).

**Table 2.** Effect of rutin (RUT), ethanol (EtOH), and cadmium (Cd) co-exposure on serum Cd level and marker enzymes of hepatic function in rats.

Groups	Cd (ppm)	GGT (U/L)	LDH (U/L)	ALP (U/L)	ACP (U/L)	AST (U/L)	ALT (U/L)
Control	0.97 ± 0.02^a^	56.16 ± 10.64^a^	11.35 ± 1.46^a^	150.5 ± 15.7^a^	17.98 ± 5.71^a^	209.94 ± 14.81^a^	54.43 ± 2.26^a^
Cd	3.25 ± 0.57^b^	127.38 ± 11.46^b^	17.95 ± 2.46^b^	222.8 ± 16.4^b^	58.35 ± 9.9^b^	208.91 ± 6.29^a^	52.64 ± 1.33^a^
Cd + EtOH	2.54 ± 0.08^b^	288.92 ± 40.12^c^	16.21 ± 1.64^b^	264 ± 9.3^c^	151.2 ± 14.53^c^	212.81 ± 0.42^a^	54.05 ± 6.25^a^
25 RUT + Cd + EtOH	1.86 ± 0.05^c^	226.39 ± 18.83^c^	15.05 ± 1.64^b^	281.6 ± 14.14^c^	110.14 ± 10.43^d^	202.08 ± 18.51^a^	51.42 ± 2.26^a^
50 RUT + Cd + EtOH	1.73 ± 0.04^d^	160.38 ± 4.09^d^	10.72 ± 1.23^a^	292.05 ± 7^c^	31.93 ± 8.82^e^	207.58 ± 9.96^a^	55.1 ± 9.84^a^
100 RUT + Cd + EtOH	1.28 ± 0.04^e^	159.8 ± 18.01^d^	9.56 ± 1.22^a^	163.35 ± 11.67^d^	20.19 ± 4.67^e^	203.65 ± 6.66^a^	54.8 ± 4.92^a^

Data are presented as the mean ± SD (*n* = 5). Data with different superscripts are significantly different (*p* < 0.05).

### MDA level and antioxidant defense system in serum, liver and kidney of Cd–EtOH intoxicated rats pretreated with RUT

**Figure 1.**
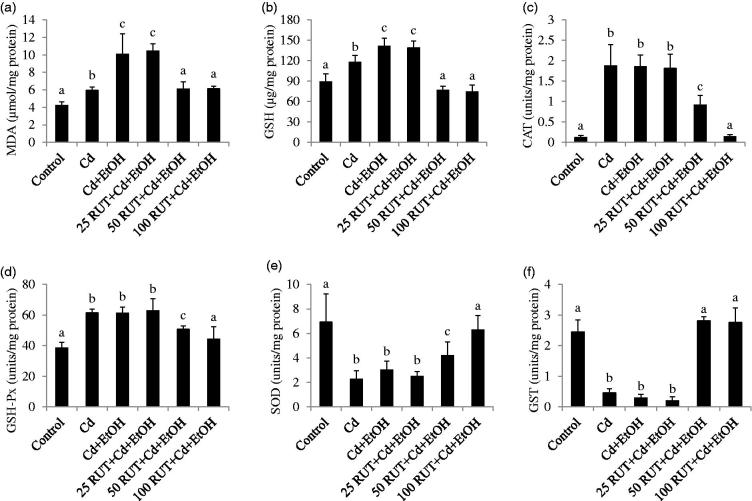
Effects of rutin (RUT), ethanol (EtOH) and cadmium (Cd) co-exposure on rat liver oxidative stress markers (a) malondialdehyde (MDA), (b) reduced glutathione (GSH), (c) catalase (CAT), (d) glutathione peroxidase (GSH-Px), (e) superoxide dismutase (SOD), (f) glutathione-*S*-transferase (GST). Data are presented as the mean ± SD (*n* = 5). Bars with different alphabets are significantly different (*p* < 0.05).

**Figure 2.**
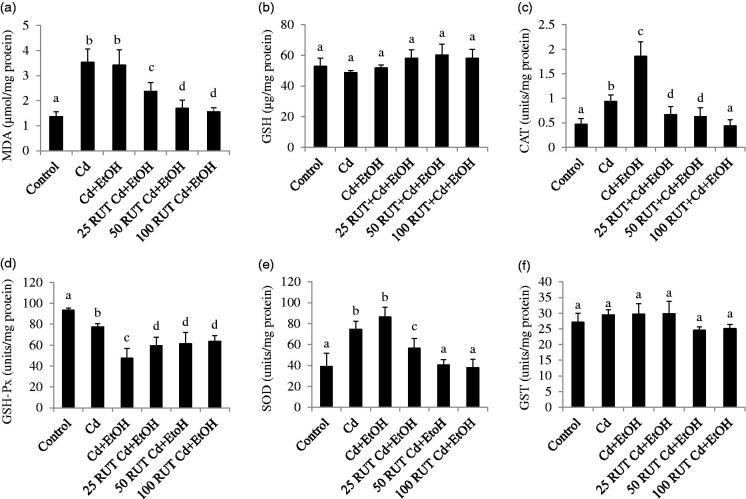
Effects of rutin (RUT), ethanol (EtOH) and cadmium (Cd) co-exposure on oxidative stress markers in rat serum (a) malondialdehyde (MDA), (b) reduced glutathione (GSH), (c) catalase (CAT), (d) glutathione peroxidase (GSH-Px), (e) superoxide dismutase (SOD), (f) glutathione-*S*-transferase (GST). Data are presented as the mean ± SD (*n* = 5). Bars with different alphabets are significantly different (*p* < 0.05).

**Figure 3.**
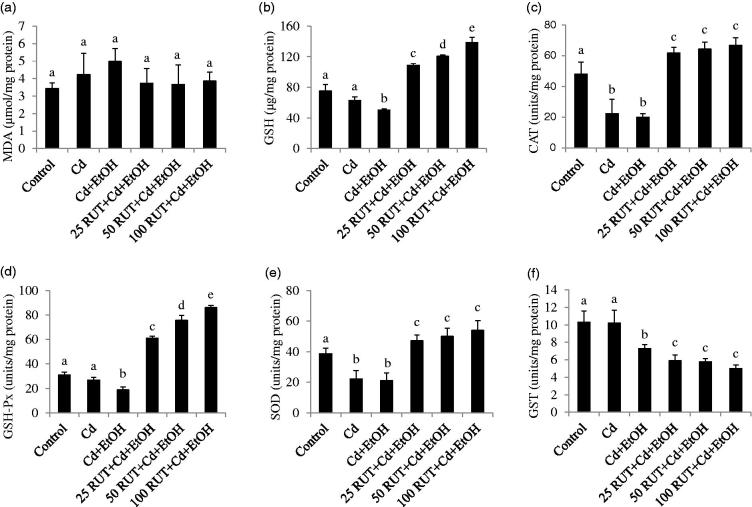
Effects of rutin (RUT), ethanol (EtOH) and cadmium (Cd) co-exposure on rat kidney oxidative stress markers (a) malondialdehyde (MDA), (b) reduced glutathione (GSH), (c) catalase (CAT), (d) glutathione peroxidase (GSH-Px), (e) superoxide dismutase (SOD), (f) glutathione-*S*-transferase (GST). Data are presented as the mean ± SD (*n* = 5). Bars with different alphabets are significantly different (*p* < 0.05).

Figures 1–3 show the antioxidant status of the liver, serum and kidney of control and treated rats. Exposure to Cd and Cd + EtOH increased the concentrations of MDA and GSH as well as CAT and GSH-Px activities in the liver but reduced the activities of hepatic SOD and GST compared to control group (*p* < 0.05). The same trend was observed in the values of hepatic MDA, GSH, CAT, GSH-Px, SOD and GST of rats treated with 25 RUT + Cd + EtOH (*p* < 0.05). More so, the increased level of MDA and GSH was more pronounced in the liver of Cd + EtOH-treated group than that of Cd alone and control animals. However, 50 and 100 mg/kg RUT restored the levels of MDA and GSH and activities of CAT, GSH-Px, SOD, and GST in liver homogenate of rats to control values (Figure 1).

Treatment of animals with Cd and Cd + EtOH increased the concentration of MDA and activities of CAT and SOD but reduced GSH-Px activity in the serum compared to control values. Interestingly, the increased serum CAT and reduced GSH-Px activities were exaggerated in the Cd + EtOH treated animals compared to the Cd treated rats. The enzymatic activities of serum CAT and SOD in 25 RUT + Cd + EtOH treated group were significantly reduced (*p* < 0.05) compared to Cd + EtOH treated animals. Treatment of animals with 50 RUT + Cd + EtOH or 100 RUT + Cd + EtOH decreased serum MDA level, CAT and SOD activities compared to Cd + EtOH groups (*p* < 0.05). The reduction in the level of serum CAT activity by RUT was more effective at 100 mg/kg body weight. Furthermore, GSH-Px activity was observed to increase in the serum of 25 RUT + Cd + EtOH, 50 RUT + Cd + EtOH and 100 RUT + Cd + EtOH treated animals compared to Cd + EtOH treated animals (*p* < 0.05). The activity of GST and GSH level in the serum were statistically unchanged across all groups. However, there was slight increase in GSH level in the 25 RUT + Cd + EtOH, 50 RUT + Cd + EtOH and 100 RUT + Cd + EtOH and insignificant decrease in GST activity in the 50 RUT + Cd + EtOH and 100 RUT + Cd + EtOH treated animals compared to other treatment groups (Figure 2).

It was observed that the increased concentration of MDA in the kidney of Cd and Cd + EtOH treated animals were not statistically significant (*p* > 0.05) compared to control values (Figure 3). The level of renal MDA was not significantly altered in the Cd + EtOH groups co-treated with the different doses of RUT (25, 50, 100 mg/kg body wt.) compared to control values. The animals exposed to Cd + EtOH showed decreased concentration of renal GSH level and enzymatic activities of CAT, GSH-Px, SOD and GST compared to control value (*p* < 0.05). Exposure to Cd alone did not significantly alter the renal level of GSH and enzymatic activities of GSH-Px and GST when compared to control values. The 25 RUT + Cd + EtOH, 50 RUT + Cd + EtOH and 100 RUT + Cd + EtOH treated animals showed increased renal GSH concentration and enzymatic activities of CAT, GSH-Px, and SOD as well as decreased activity of GST in a dose-dependent manner compared to other experimental groups (Figure 3).

### Concentrations of serum markers for renal and hepatic functions

**Figure 4.**
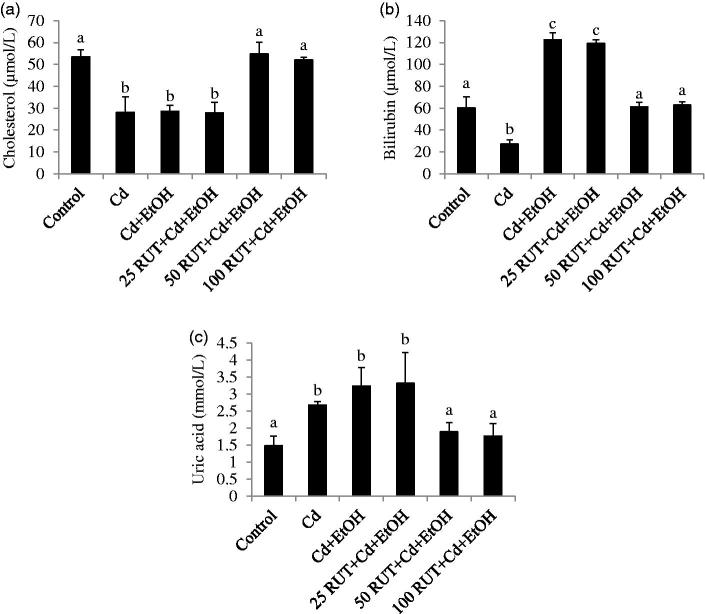
Effects of rutin (RUT), ethanol (EtOH) and cadmium (Cd) co-exposure on serum (a) cholesterol level (b) bilirubin level and (c) uric acid level in rats. Data are presented as the mean ± SD (*n* = 5). Bars with different superscript are significantly different (*p* < 0.05).

Figure 4 shows the concentration of cholesterol, bilirubin and uric acid in serum of control and treated animals. The level of serum cholesterol in Cd, Cd + EtOH and 25 RUT + Cd + EtoH treated animals was significantly decreased compared to control values (*p* < 0.05). Co-treatment with RUT at 50 and 100 mg/kg restored the cholesterol level to control values. Serum bilirubin level was significantly higher in the control group compared to Cd-treated animals (*p* < 0.05). The serum of Cd + EtoH and 25 RUT + Cd + EtoH treated animals showed significantly increased level of bilirubin as compared to control (*p* < 0.05). Supplementation with 50 and 100 mg/kg RUT decreased serum bilirubin level to control values. Furthermore, elevated level of serum uric acid was observed in Cd, Cd + EtOH and 25 RUT + Cd + EtOH treated animals as compared to the control. Co-treatment with 50 and 100 mg/kg RUT significantly restored the level of uric acid to control values.

### Histopathology of rat liver and kidney

**Figure 5.**
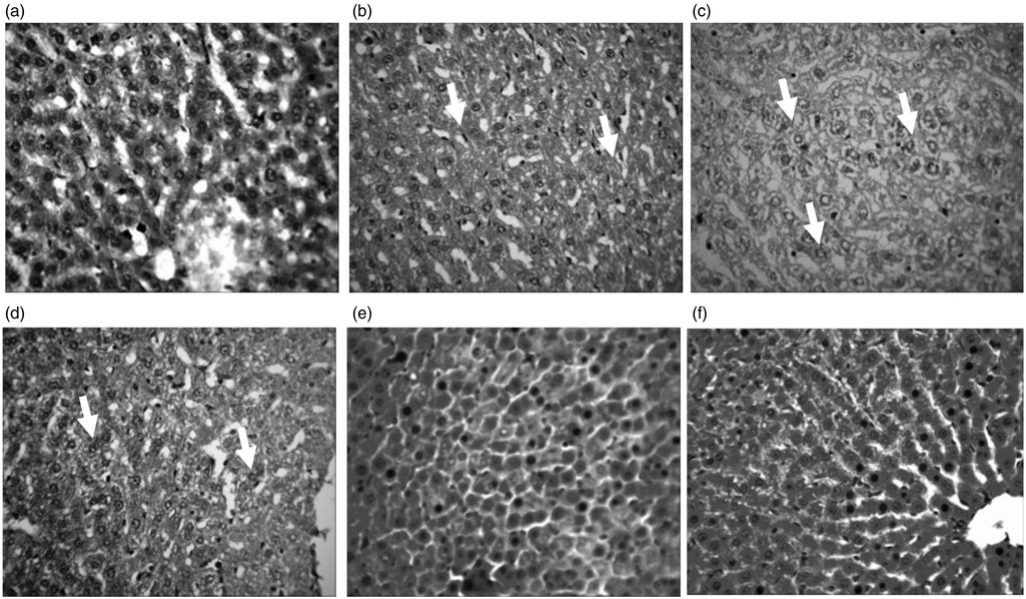
Representative photomicrographs from the liver showing the protective effect of RUT against Cd + EtOH-induced hepatic injury in rats. (a) Control: No visible lession seen (b) Cd: There is mild diffuse degeneration of hepacytes (arrows). (c) Cd + EtOH: There is a severe diffuse hydropic degeneration of hepatocytes (arrows). (d) 25 RUT + Cd + EtOH: Severe central venous congestion and periportal cellular infiltration by mononuclear cells and degeneration of hepatocytes (arrows). (e) 50 RUT + Cd + EtOH: No visible lesion seen (F) 100 RUT + Cd + EtOH: No visible lesion seen. H & E; mag ×400.

**Figure 6.**
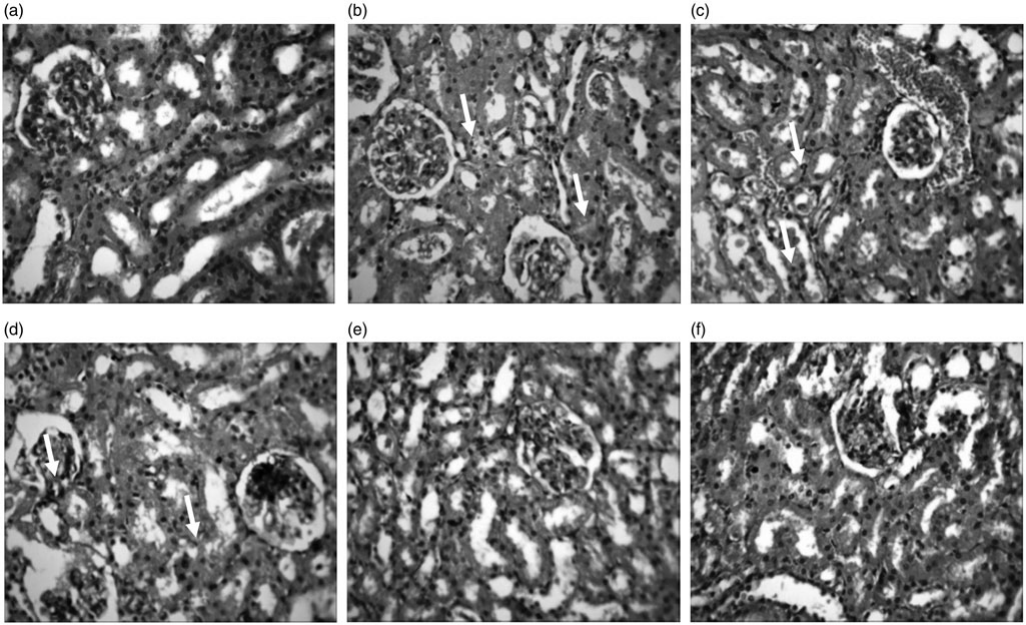
Representative photomicrographs from the kidney showing the protective effect of RUT against Cd + EtOH-induced renal injury in rats. (a) Control: No visible lesions seen (b) Cd: There is a diffuse tubular and glomerular degeneration (small arrows). (c) Cd + EtOH: Severe renal cortical congestion and diffuse tubular and glomerular degeneration (small arrows). (d) 25 RUT + Cd + EtOH: severe glomerular degeneration and necrosis (arrows). (e) 50 RUT + Cd + EtOH: No visible lesions seen (f) 100 RUT + Cd + EtOH: No visible lesions seen. H & E; mag ×400.

Figures 5 and 6 shows the representative photomicrographs of liver and kidney sections of control and treated rats. The histology of liver sections of control, 50 RUT + Cd + EtOH and 100 RUT + Cd + EtOH animals revealed no visible lesion (Figure 5). In contrast, treatment with Cd resulted in mild hydropic degeneration of hepatocytes while treatment with Cd + EtOH showed severe hydropic degeneration of hepatocytes. Furthermore, the liver of 25 RUT + Cd + EtOH treated animals showed severe central venous congestion and periportal cellular infiltration by mononuclear cells alongside hydropic degeneration of hepatocytes (Figure 5). The morphology of kidney sections for control animals were normal and showed no visible lesions. The kidney sections of rats exposed to Cd showed diffuse tubular and glomerular degeneration. There were also severe congestion of the renal cortex, degeneration and necrosis of glomerulus in kidney sections of Cd + EtOH and 25 RUT + Cd + EtOH treated rats. However, the morphological architecture of the kidney sections of 50 RUT + Cd + EtOH and 100 RUT + Cd + EtOH treated animals appeared normal with no visible lesions.

## Discussion

The present study demonstrates that RUT has protective effects against Cd + EtOH induced oxidative damage in hepatic and renal tissues as well as in the blood of experimental rats. The body weights of all rats increased across all groups at the end of the present study indicating the absence of general toxicity. This observation has been reported previously in Cd and ethanol treated rats (Brzóska et al. 2013). Accordingly, the absolute and relative weights of the liver of Cd intoxicated rats were statistically unchanged, but a decrease in the absolute and relative weights of the kidney and liver following combined treatment of Cd and EtOH was noted at the end of the study. Tissue mass is a balance between proliferation, death and differentiation and a shift in equilibrium towards apoptosis can reduce cell mass (Lockshin and Zakeri 2007). Thus, the observed effect on kidney and liver weights in our study can be attributed to pathological changes and decreased tissue mass due to Cd + EtOH induced oxidative damage of renal and hepatic tissues (Wang et al. 2014). The liver and kidney are recognized as important organs affected by Cd and EtOH metabolism. In the liver, Cd binds to sulfhydryl groups of critical proteins where it alters the mitochondrial membrane transition and promotes hepatic injury through leakage of superoxide anions (Matović et al. 2015). Indeed, Cd-metallothionein complex formed in the liver gets to the kidney where it is filtered through the glomerulus and reabsorbed by the proximal tubular cells. The proteases in the lysosomes of proximal tubular cells degrade the complex releasing Cd to cause kidney damage (Salińska et al. 2013). Treatment of Cd + EtOH rats with 100 mg/kg RUT restored the absolute and relative weights of liver and kidney close to control values. The antioxidant protective properties of RUT might be responsible for these positive effects of RUT on the kidney and liver (Abarikwu et al. 2013; Aruna et al. 2014; Nafees et al. 2015).

It is known that Cd accumulates in serum and organs of rats after Cd treatment and that EtOH decreases the absorption of Cd from the gastrointestinal tract (Brzóska et al. 2013). Consistent with our present findings, there was reduced serum Cd level in Cd + EtOH treated animals compared to Cd treated animals. The decrease in Cd burden due to concomitant EtOH exposure is not indicative of reduced health risk as EtOH may enhance Cd toxicity by disrupting the body status of essential elements (Brzóska et al. 2013). The dose-dependent decrease in the Cd level caused by RUT treatment as compared to the values found in Cd-treated animals has been reported previously by us (Abarikwu et al. 2017) and further supports the metal chelating potentials of antioxidant flavonoids (Flora and Pachauri 2010).

The elevation of MDA concentration accompanied by a concomitant increase and/or decrease in activities of antioxidant defence systems such as CAT, SOD, GSH-Px and GST are indicative of oxidative stress in tissues (Cinar et al. 2000; Dabak et al. 2009; Gałażyn-Sidorczuk et al. 2012; Kamoun et al. 2017). The alterations in liver, serum and kidney oxidative status due to Cd and/or Cd + EtOH exposure observed in the present study also corroborated previous findings (Sharma et al. 1991; Jurczuk et al. 2004), and are attributed to the increased generation of reactive oxygen species and/or decreased level of antioxidants required to protect tissues against oxidative damage (Gałażyn-Sidorczuk et al. 2012; Dkhil et al. 2014; Matović et al. 2015). The ability of RUT at higher doses (50, 100 mg/kg body wt.) to protect against increased MDA level and the fluctuation in antioxidant defence system in liver, serum and kidney tissues is due to the scavenging of the free radicals that initiates the chain process of lipid peroxidation (Nafees et al. 2015). In the liver tissue of the present study, hepatic injuries caused by Cd + EtOH were associated with an elevation of serum ALP, GGT, ALP and ACP activities whereas renal injuries were confirmed with an elevation of uric acid. These markers are important predictors for disturbances in the integrity of cell membrane (Chuffa et al. 2014; Kwo et al. 2017). Several studies (Pari and Karthikesan, 2007; Dkhil et al. 2014) have also confirmed our present findings. In most of the published reports, the alterations in serum markers for renal and hepatic functions were associated with altered liver architecture, hepatocellular damage and renal damage (Dkhil et al. 2014). One possible explanation to this is that the supra-normal level of reactive oxygen species resulting from Cd and EtOH metabolism leads to cell membrane lipid peroxidation, resulting to increased membrane permeability and consequent release of these marker enzymes from tissue into the bloodstream (Chuffa et al. 2014). The administration of RUT (50, 100 mg/kg body wt.) decreased the activities of the serum enzymes, as well as bilirubin and uric acid levels, confirming the hepato- and nephro-protective effects of RUT in the present study. Several flavonoids including quercetin, proanthocyanidins, caffeic acid, etc., that occurs naturally in fruits have also been reported to protect the liver and kidney from damage mediated through oxidative stress (Pari and Karthikesan 2007; Seiva et al. 2012; Nazima et al. 2014). This supports our present findings. The inability of the low-dose RUT (25 mg/kg) to normalize the levels of these marker enzymes confirmed that the protective effects of RUT on tissues is dependent on the dose, and that the dose required to protect renal and hepatic tissues from damage due to peroxidative injury might be different from the dose that chelate metals *in vivo* (Abarikwu et al. 2016). The 7- and 5-hydroxyl groups, o-dihydroxy structure of ring B, and the 2′ 3′ double bond in conjugation with a 4-oxo function are features proposed to be responsible for the antioxidant potential of RUT in most experimental models (Russo et al. 2000).

## Conclusions

The present study demonstrated that RUT was able to decrease the risk of hepatic and renal dysfunction by reducing cell membrane peroxidative injury, serum bilirubin, cholesterol, and uric acid levels and modulates the enzyme biomarkers for tissue toxicity in rats exposed to both Cd and EtOH. Furthermore, the therapeutic effects are dose-dependent and are contributed partly by the antioxidant potential of RUT.
